# Differences in reporting food insecurity and factors associated with differences among Latino fathers and mothers

**DOI:** 10.1186/s12889-021-10971-x

**Published:** 2021-05-13

**Authors:** Sayaka Nagao-Sato, Stephanie Druziako, Aysegul Baltaci, Alejandro Omar Peralta Reyes, Youjie Zhang, Ghaffar Ali Hurtado Choque, Marla Reicks

**Affiliations:** 1 Department of Food Science and Nutrition, University of Minnesota, 1334 Eckles Ave., St. Paul, MN 55304 USA; 2 Extension Center for Family Development, University of Minnesota, 1420 Eckles Ave., St. Paul, MN 55304 USA; 3 Department of Child and Adolescent Health and Social Medicine, School of Public Health, Medical College of Soochow University, 199 Ren Ai Road, Jiangsu 215123 Suzhou, China; 4 School of Public Health, University of Maryland, 4200 Valley Dr. 1142E, College Park, MD 20742 USA

**Keywords:** Food insecurity, Difference in perceptions of parents, Latino fathers and mothers

## Abstract

**Background:**

Food security status has been assessed as a representative score for households; however, different members in the same household may perceive and report food insecurity differently. A high prevalence of food insecurity has been reported among Latino households, therefore understanding differences in reporting food insecurity by Latino father-mother dyads may improve accuracy of assessment and plans to address food insecurity. This study aimed to 1) determine demographic characteristics and/or food-related factors associated with perceptions of food security status among Latino father-mother dyads, and 2) identify factors associated with discordance in perceptions of food insecurity between dyads.

**Methods:**

Baseline data were used from a community-based, youth obesity prevention program among Latino families (*n* = 106 father-mother dyads). Food security was assessed with a 2-item food insecurity screen. Logistic regression models were used to evaluate associations between reporting food security status and predictor variables for fathers, mothers, and dyad-discordant responses.

**Results:**

Food insecurity was reported by 39% of fathers and 55% of mothers. Adjusted odds of reporting food insecurity were significantly higher for fathers perceiving their neighborhood was unsafe vs. safe (*OR*: 3.7, *p* < 0.05) and reporting lower vs. higher household income (*OR*: 3.2, *p* < 0.05). Adjusted odds of reporting food insecurity were significantly higher for mothers perceiving their neighborhood was unsafe vs. safe (*OR*: 4.1, *p* < 0.01) and reporting lower vs. higher home availability of fruit and vegetable (*OR*: 5.5, *p* < 0.01). Dyad discordance in reporting food security status occurred in 24% of the dyads. Adjusted odds of dyad discordant reports of food insecurity status were significantly higher for dyads reporting discordant responses regarding previous nutrition education (*OR*: 3.4, *p* < 0.05) and higher home fruit and vegetable accessibility (*OR*: 3.1, *p* < 0.05) compared to dyads reporting concordant responses. Among the 28 dyads who reported discordant nutrition education participation, 21 reported that fathers had never participated but mothers had participated more than once.

**Conclusions:**

Differential factors were associated with reporting food security among Latino father-mother dyads. Nutrition education for fathers that improves awareness of home food supplies and a better understanding of how food accessibility influences maternal perceptions may improve dyad discordance in reporting household food security.

## Background

Food security status, defined as access to enough food for an active, healthy life, has frequently been assessed and viewed as a representative score for the household [1]. However, the extent that all members of the household experience food insecurity may differ depending on their perception of adequacy and quality of household food stores, and worry or anxiety about home food availability [2]. Some studies indicated that females have reported higher levels of food insecurity than males [3–5]. Higher food insecurity in female-led versus male-led households was attributed to women’s socioeconomic disadvantages in female-led households, such as lower income or more dependents [4]. Differences in measurement of food insecurity between mother and father pairs in a qualitative study were based on different interpretations of terms such as “household”, “balanced meal”, and “worry” used in questions to assess food insecurity [6]. Within male and female pairs in the same household in Bangladesh, discordant responses to food security questionnaires were observed in 19% of pairs [7]. In another study, couple discordance has been reported for additional diverse household and individual characteristics such as income and marital length [8].

The reported prevalence of food insecurity among Hispanic households in the U.S. based on 2019 data from the Current Population Survey’s Food Security Supplement was 15.6%, which was higher than the 10.5% prevalence reported for U.S. households overall [9]. The prevalence of food insecurity among U.S. Hispanic households was also significantly increased from 1999 to 2000 to 2015–2016 based on National Health and Nutrition Examination Survey (NHANES) data [10]. Food insecurity is an important issue for Latino youth and adults, because of associations with inadequate nutrient intake [11–14], under-consumption of healthy foods [14], and a high prevalence of obesity [15–17]. Latinos play a significant role in the U.S. economy accounting for 17% of the labor force [18], often working several jobs to attain financial gains. Income and other social determinants of health such as lack of access to jobs, inadequate transportation and wages, and inequities in assets and wealth may contribute to the disproportionately high rates of food insecurity among Latinos. Eligible immigrant families, in particular, may not access food assistance to address food insecurity because of immigration policy changes and enforcement activities [19]. Federal food and nutrition assistance programs and community-based nutrition education have been used to address food insecurity leading to positive outcomes regarding food security status [20–22].

Previous studies with Latino youth and adults as well as other racial/ethnic groups have identified a variety of factors influencing household food insecurity. These include demographic and economic characteristics such as sex, marital status, educational attainment, acculturation, household income, and employment status [4, 23, 24]. Home food environment and other environmental factors were also reported, including social support from family members, home food availability, home food accessibility [24–27], and neighborhood safety and geographic location [23, 28]. In addition, discordant perceptions of food security in the same households were observed between Latino parents and adolescents where discordance was associated with parent-adolescent conflict, presence of a female adolescent and household income [24].

Different perceptions and experiences regarding food insecurity by members in the same households could lead to inaccurate assessment of food security status. Therefore, if one parent in the household perceives a higher level of household food security than another parent, they may underestimate the prevalence when reporting for the household, which could impact eligibility for food and nutrition assistance programs and underestimate federal and state level prevalence information. Awareness of the differences between father-mother dyad reports of food insecurity and factors accounting for these differences could help those who plan food and nutrition assistance programs to better address food insecurity.

To date, differences among Latino father-mother dyads in reporting food security status have not been reported. The purpose of this study was to 1) determine demographic characteristics and/or food-related factors associated with perceptions of food insecurity status among Latino father-mother dyads in the same households, and 2) identify those factors correlated with discordance in perceptions of food insecurity between the dyads.

## Methods

### Design and setting

The current study used cross-sectional baseline data from a community-based intervention program to improve paternal parenting practices and youth energy balance-related behaviors (Padres Preparados, Jóvenes Saludables) [29]. Latino fathers or male caregivers (hereafter referred to as fathers) and early adolescents (10–14 years) were the primary research participants, however mothers or female caregivers (hereafter referred to as mothers) were also invited to attend educational sessions and complete evaluation data collection procedures as part of the intervention. Baseline data collection occurred from 2017 to 2020 at three churches and two Latino-serving non-profit community centers in the Minneapolis/St. Paul metropolitan area.

### Participants

Participants were recruited using flyers, word of mouth, and announcements at community events. Eligibility criteria for fathers included self-identifying as Latino, speaking Spanish, and having meals with the youth participant at least three times in a week. Father-adolescent dyads were the primary research participants; however, mothers were also welcome to attend the same data collection and educational sessions with fathers. Eligibility criteria for mothers included self-identifying as Latina, speaking Spanish and as spouses/partners of fathers who were parents of the youth participants. Eligibility criteria for mothers did not include frequency of meals with the youth participant; however, meal frequency with mothers was higher than meal frequency with fathers (58% of mothers vs. 42% of fathers had meal frequency ≥ 7 times/week, *p* = 0.02) according to results from the baseline survey. Three hundred sixty-nine families were interested in the Padres Preparados, Jóvenes Saludables study, and 277 families remained after screening for eligibility. Baseline data, which included sociodemographic information, height and weight, and questionnaire data regarding food-related activities and the home food environment were analyzed in the current study from 106 father-mother dyads where both had completed food security questions. All survey questions were first written in English (unless tested, valid questions or scales were available in Spanish), translated into Spanish by research team members whose native language was Spanish, and back translated into English to confirm the accuracy of the translation. The research team was comprised of primarily native Spanish speaking individuals who came to consensus after discussion about how to structure questions and also reviewed questions for potential understanding by participants. After completion of the questionnaire, fathers received cash compensation ($35) for participating in baseline data collection procedures. Fathers and mothers provided informed consent prior to participation. The study was approved by the University of Minnesota Institutional Review Board.

### Measures

#### Anthropometric measures

Trained researchers measured body weight and height of fathers and mothers using a digital scale (BWB-800 Scale, Tanita) and a stadiometer based on standard procedures [30]. Measurements were completed twice to the nearest 0.1 kg weight and 0.1 cm for height. Body mass index (BMI) was calculated using the average weight and height (kg/m^2^).

#### Sociodemographic characteristics

Fathers and mothers individually reported their age, educational attainment, employment status, household income, number of years in the U.S., language spoken at home, and marital status. Age, educational attainment, employment status, and household income were dichotomized as < median age vs. ≥ median age, < high school vs. ≥ high school, full-time/self-employed vs. part-time/not-employed, household income < $25,000 vs. ≥ $25,000. Acculturation score group was based on the number of years in the U.S. (coded as 0 = < 10 years, 1 = ≥10 and < 20, 2 = ≥20 and < 30, 3 = ≥30) and language spoken at home (coded as 0 = only native or more native language than English, 1 = English equal to native language, 2 = only English or more English than native language) [31]. A clarification was included with this question indicating that native languages were Spanish or any language other than English. The acculturation score group was the sum of the coded responses for number of years in the US and language spoken at home and was categorized as low (0–1 point), middle (2 points), or high acculturation score group (3–5 points). In a previous study, the acculturation score group was associated with education, income, and health insurance among Hispanic adults [31].

#### Food security

Food security was assessed using a 2-item food insecurity screen in Spanish. The screening tool used 2-items from the US Department of Agriculture 18-item Household Food Security Survey [1], including “Within the past 12 months, we worried about whether our food would run out before we got money to buy more” and “Within the past 12 months, the food we bought just didn’t last and we didn’t have money to get more”, with response options of often true or sometimes true vs. never true. Hager et al. reported that an affirmative response (often true or sometimes true) to at least one of these two questions showed 97% sensitivity and 83% specificity for detection of household food insecurity [32].

#### Participation in food assistance programs

Current participation in any of the following food or financial assistance programs was assessed (yes/no): Special Supplemental Nutrition Program for Women, Infants & Children (WIC), Supplemental Nutrition Assistance Program-Education (SNAP-Ed), free or reduced-price meals at school, and the Minnesota Family Investment Program. Ever having participated in any of the following nutrition education programs was assessed (yes/no): SNAP-Ed, Expanded Food and Nutrition Education Program (EFNEP), WIC, and Cooking Matters.

#### Physical home food environment

Questions about home food availability and accessibility were adapted from a previous cross-sectional study of adolescents (Project EAT - Eating Among Teens) [33]. Home food availability was assessed by summing coded responses to six questions regarding availability of fruits, vegetables, junk foods, soda pop, sweets, and potato chips (coded as 1 = never, 2 = sometimes, 3 = usually, 4 = always) [33, 34]. Scores for fruit and vegetable (FV) availability were the sum of the coded responses to the questions about availability of fruits and vegetables. Home FV accessibility was assessed by summing coded responses for three questions regarding accessibility of FV (coded as 0 = never, 1 = sometimes, 2 = usually, 3 = always) [33, 35]. Summed coded responses were dichotomized as < median value vs. ≥ median value. Project EAT variable information was reported with α = 0.61 for home FV availability and α = 0.78 for home FV accessibility among a study population including 3.5% Hispanic participants [35].

#### Meal frequency with the youth participant

A variable of meal frequency with the youth program participant (coded as 0 = never, 1 = 1–2 times, 2 = 3–4 times, 3 = 5–6 times, 4 = 7 times, 5 = > 7 times) was adapted from Project EAT [36]. This variable was a family meal patterns variable with α = 0.78 in a sample population including 3.5% Hispanic participants [33].

#### Environmental factors

Neighborhood safety was assessed based on agreement with two questions adapted from Saelenes et al. [37] regarding perceptions of whether the crime rate makes walking in their neighborhood unsafe during the day and at night (coded as 1 = strongly disagree to 4 = strongly agree). Neighborhood safety was described as a crime safety variable with test-retest reliability *r* = 0.80 in a sample population which was 9.3% Hispanic/Latino [37]. Family stress was assessed using three questions about the importance of family relations, conflict between personal and family goals, and individualism among family members (coded as 1 = not at all worried to 5 = extremely worried). This measure was adapted from Cervantes et al. as a cultural/family conflict variable with test-retest reliability of *r* = 0.86 in a Hispanic population [38]. These variables were represented by the sum of the coded responses, dichotomized as < median value vs. ≥ median value.

### Statistical analysis

Demographic data were described using means and standard deviations (mean ± SD) or frequencies for categories for fathers and mothers. To determine the differences in demographic characteristics between fathers and mothers, *t*-test or Fisher’s exact tests were conducted.

Associations between food security and predictor variables were examined among fathers and mothers separately using logistic regression models. Preliminary analyses were conducted to assess appropriateness of variables for inclusion in regression models. Fisher’s exact tests were used to determine differences in sociodemographic characteristics, Participation in food assistance programs, physical home food environment, and environmental factors by food security status. Predictor variables that showed differences by either fathers’ or mothers’ report of food insecurity were considered for inclusion in the regression models (*p* < 0.15), including neighborhood safety, household income, current participation in food assistance programs, home FV availability, and family stress. Interactions between predictor variables were tested. The logistic regression models were created for fathers and mothers separately. In the models, food security status served as the dependent variable. Neighborhood safety, household income, current participation in food assistance programs, home FV availability, and family stress were included as independent variables based on the preliminary analyses and tests for multicollinearity.

A cross-tabulation of household food security, as reported by fathers and mothers, was examined to evaluate concordance within dyads using Fisher’s exact test. Father and mother dyads were dichotomized into perceived concordance or discordance regarding food security status. Concordance was defined as dyads where both the father and mother reported food security status or both the father and mother reported food insecurity status. The predictors for dyad concordance in reports of food security status were determined using concordance or discordance with distinct characteristics of the variables within dyads. Fisher’s exact tests were used to determine appropriateness of covariates for inclusion in the logistic regression model. Predictors that showed differences by dyads reported food security status were considered for inclusion in the regression models (*p* < 0.15), including home food availability, home FV accessibility, acculturation score group, and ever participated in nutrition education. Interactions between predictor variables were tested showing that home food availability was significantly associated with home FV accessibility, therefore, the home food availability variable was not included in the model. Logistic regression analysis was used to evaluate the predictors for discordance in reports of food security within dyads.

The level of significance was set at *p* < 0.05. Statistical analysis was performed using R version 3.5.1 and SPSS for Windows, version 26.

## Results

### Participant characteristics

Similarities among fathers and mothers were observed regarding education level and household income (Table 1). About one-third had less than a high school education and about one-half had a household income less than $25,000. Significant differences were observed between fathers and mothers in age (fathers were slightly older than mothers), BMI (mothers had slightly higher BMIs than fathers), employment status (more fathers were employed full time than mothers), and acculturation score group (more mothers were in the lower acculturation score group than fathers) (all *p* < 0.05) (Table 1). Of participants, 93% of fathers and 91% of mothers reported being married.

**Table 1:** Demographic characteristics and food security status of 106 father-mother dyads

		Father	Mother	*p*
*n*		106	106	
Age, mean ± SD		43.0 ± 7.6	40.1 ± 6.4	< 0.01
BMI, mean ± SD		29.5 ± 3.4	31.2 ± 5.2	0.01
Educational attainment, *n* (%)	< High school	36 (34)	38 (37)	0.77
	≥ High school	69 (65)	66 (63)	
Employment status, *n* (%)	full/self-employed	90 (88)	46 (45)	< 0.01
	not full/self-employed	12 (12)	56 (55)	
Household income, *n* (%)	< $25,000	41 (41)	41 (45)	0.66
	≥ $25,000	59 (59)	50 (55)	
Acculturation score group, *n* (%)	Low	42 (41)	71 (69)	< 0.01
	Middle	44 (43)	23 (22)	
	High	17 (16)	9 (8)	
Marital status, *n* (%)	Married	96 (93)	95 (91)	0.80
	Not married	7 (7)	9 (9)	
Food security, *n* (%)	Food insecure	41 (39)	58 (55)	0.03
	Food secure	65 (61)	48 (45)	

*p*-value derived by *t*-test for age and BMI and Fisher’s exact test for educational attainment, employment status, household income, acculturation score group, marital status, and food security. *SD* Standard deviation, *BMI* Body mass index

Approximately half of participants reported being food insecure (39% for fathers and 55% for mothers) with significantly more mothers than fathers reporting food insecurity (*p* < 0.05) (Table 1). The majority of dyads reported concordant food security or insecurity status (76% of dyads, *n* = 81), while reports from 25 dyads were discordant. In the dyads who reported concordant food security status, 44 dyads reported food security, and 37 dyads reported insecurity. In the dyads who reported discordant food security status, 21 mothers reported food insecurity status while fathers did not and four fathers reported food insecurity status while mothers did not.

### Factors associated with reporting food insecurity by fathers and mothers

The number of both mothers and fathers perceiving their neighborhood to be safe vs. unsafe differed by reports of food security or insecurity status (Table 2). Household income and current participation in food assistance programs differed significantly between fathers who reported food security and food insecurity (*p* < 0.05). Home FV availability and family stress differed between mothers who reported food security vs. food insecurity (*p* < 0.05) (Table 2). After assessment for appropriateness of variables for inclusion in regression models, neighborhood safety, household income, current participation in food assistance programs, home FV availability, and family stress were included as covariates in the logistic regression models (Table 3). Adjusted odds of reporting food insecurity were significantly higher for fathers who perceived their neighborhood was unsafe vs. safe (odds ratio (*OR*): 3.7) and had a lower vs. higher household income (*OR*: 3.2). Adjusted odds of reporting food insecurity were significantly higher for mothers who perceived their neighborhood was unsafe vs. safe (*OR*: 4.1) and reported lower vs. higher home availability of FV (*OR*: 5.5).

**Table 2:** Frequency of food security status by predictor variables (106 Latino fathers and mothers)

Predictor variables		Fathers			Mothers		
		Food secure (*n* = 65) *n* (%)	Food insecure (*n* = 41) *n* (%)	*p*	Food secure (*n* = 48) *n* (%)	Food insecure (*n* = 58) *n* (%)	*p*
Age	Younger	24 (37)	16 (39)	1.00	24 (50)	25 (43)	0.56
	Older	40 (62)	25 (60)		24 (50)	33 (56)	
Educational attainment	< High school	20 (31)	16 (39)	0.53	19 (39)	19 (33)	0.68
	≥ High school	44 (68)	25 (60)		29 (60)	37 (66)	
Employment status	Full-time	59 (90)	31 (83)	0.35	24 (51)	22 (40)	0.32
	Not full-time	6 (9)	6 (16)		23 (48)	33 (60)	
Household income	< $25,000	17 (28)	24 (60)	< 0.01	17 (40)	24 (48)	0.53
	≥ $25,000	43 (71)	16 (40)		25 (59)	25 (51)	
Acculturation score group	Low	26 (26)	16 (25)	0.86	33 (33)	38 (79)	0.37
	Middle	25 (25)	19 (30)		9 (9)	14 (29)	
	High	11 (11)	6 (9)		6 (6)	3 (6)	
Current participation in food assistance programs^a^	Never	42 (64)	17 (41)	0.03	25 (52)	25 (44)	0.56
	≥ 1 time	23 (35)	24 (58)		23 (47)	31 (55)	
Ever participated in nutrition education^b^	Never	32 (49)	13 (33)	0.15	16 (34)	13 (23)	0.27
	≥ 1 time	33 (50)	26 (66)		30 (65)	43 (76)	
Home food availability^c^	Lower availability	27 (42)	21 (56)	0.22	12 (26)	20 (36)	0.29
	Higher availability	37 (57)	16 (43)		34 (73)	35 (63)	
Home FV availability	Lower availability	27 (41)	18 (46)	0.69	15 (31)	36 (64)	< 0.01
	Higher availability	38 (58)	21 (53)		33 (68)	20 (35)	
Home FV accessibility	Lower accessibility	20 (31)	12 (30)	1.00	12 (26)	23 (40)	0.15
	Higher accessibility	43 (68)	27 (69)		34 (73)	34 (59)	
Neighborhood safety	Safe	34 (53)	10 (26)	0.01	27 (56)	18 (31)	0.02
	Unsafe	29 (46)	28 (73)		21 (43)	39 (68)	
Family stress	Less stress	35 (54)	16 (39)	0.16	25 (52)	16 (28)	0.02
	More stress	29 (45)	25 (60)		23 (47)	41 (71)	

*p*-value derived by Fisher’s exact test

^a^ WIC, SNAP-Ed, free or reduced-price meals at school, and the Minnesota Family Investment Program; ^b^ SNAP-Ed, EFNEP, WIC, and Cooking Matters; ^c^ Food included fruits, vegetables, junk foods, soda pop, sweets, and potato chips

*FV* fruit and vegetable, *WIC* Special Supplemental Nutrition Program for Women, Infants & Children, *SNAP-Ed* Supplemental Nutrition Assistance Program-Education, *EFNEP* Expanded Food and Nutrition Education Program

**Table 3:** Associations between food insecurity status and household environmental factors (106 Latino father-mother dyads)

	Adjusted odds ratio [95% confidence interval]	
Predictors of food insecurity	Fathers (*n* = 92)	Mothers (*n* = 88)
Neighborhood safety (ref; safer)	3.7* [1.3, 10.3]	4.1** [1.5, 11.4]
Household income (ref; > = $25000)	3.2* [1.2, 8.2]	1.6 [0.6, 4.2]
Current participation in food assistance programs^a^ (ref; at least 1 program)	2.1 [0.8, 5.5]	1.9 [0.7, 5.2]
Home FV availability (ref; higher)	1.1 [0.4, 3.0]	5.5** [2.0, 15.4]
Family stress (ref; less stress)	2.0 [0.7, 5.2]	2.4 [0.9, 6.5]
Cox-Snell R^2^	0.21	0.25

^a^ WIC, SNAP-Ed, free or reduced-price meals at school, and the Minnesota Family Investment Program; * *p* < 0.05, ** *p* < 0.01

*FV* fruit and vegetable, *SNAP-Ed* Supplemental Nutrition Assistance Program-Education, *WIC* Special Supplemental Nutrition Program for Women, Infants & Children

### Factors associated with dyad discordance in reporting food security status

Differences within dyads in reported acculturation score group, ever participated in nutrition education, home food availability, and home FV accessibility tended to be associated with dyad discordance in reporting food security status compared with other variables (*p* < 0.1) (Table 4). After assessment for multicollinearity, dyad discordances in reported acculturation score group, ever participated in nutrition education, and home FV accessibility were included in logistic regression models as covariates. Adjusted odds of discordant dyad reports of food insecurity were significantly higher for dyads with discordant responses regarding previous nutrition education and home FV accessibility (*OR*: 3.4 and 3.1, respectively), compared to dyads who reported concordant responses regarding previous nutrition education and home FV accessibility (Table 5). Twenty-eight father-mother dyads reported discordance in ever participated in nutrition education. Among them, 21 dyads reported that fathers had never participated in nutrition education but mothers participated more than once.

**Table 4:** Frequency of concordance/discordance in predictor variables by concordance/discordance in dyad-reported food security (106 Latino dyads)

Predictor variables: categories	Concordance in predictor variables	Dyads reported food security status^a^		
		Discordant (*n* = 25) *n* (%)	Concordant (*n* = 81) *n* (%)	*p*
Age: younger or older	Discordant	4 (16)	21 (25)	0.42
	Concordant	20 (83)	60 (74)	
Educational attainment: < high school or ≥ high school	Discordant	7 (29)	32 (40)	0.35
	Concordant	17 (70)	47 (59)	
Employment status: not full-time or full-time	Discordant	12 (52)	39 (51)	1.00
	Concordant	11 (47)	37 (48)	
Household income: < $25,00 or ≥ $25,000	Discordant	7 (35)	16 (23)	0.39
	Concordant	13 (65)	51 (76)	
Acculturation score group: low, middle, or high	Discordant	13 (56)	26 (33)	0.06
	Concordant	10 (43)	51 (66)	
Current participation in food assistance programs^b^: never or ≥ 1 time	Discordant	9 (37)	18 (22)	0.18
	Concordant	15 (62)	62 (77)	
Ever participated in nutrition education^c^: never or ≥ 1 time	Discordant	11 (44)	17 (22)	0.07
	Concordant	14 (56)	58 (77)	
Home food availability^d^: lower or higher availability	Discordant	14 (58)	25 (34)	0.06
	Concordant	10 (41)	47 (65)	
Home FV availability: lower or higher availability	Discordant	8 (33)	23 (29)	0.80
	Concordant	16 (66)	55 (70)	
Home FV accessibility: lower or higher accessibility	Discordant	9 (39)	15 (19)	0.09
	Concordant	14 (60)	61 (80)	
Neighborhood safety: safe or unsafe	Discordant	7 (28)	25 (32)	0.81
	Concordant	18 (72)	51 (67)	
Family stress: less or more stress	Discordant	10 (43)	33 (40)	0.82
	Concordant	13 (56)	48 (59)	

*p*-value derived by Fisher’s exact test

^a^ Concordance was defined as dyads where both the father and mother reported food security status or both the father and mother reported food insecurity status; ^b^ WIC, SNAP-Ed, free or reduced-price meals at school, and the Minnesota Family Investment Program; ^c^ SNAP-Ed, EFNEP, WIC, and Cooking Matters; ^d^ Food included fruits, vegetables, junk foods, soda pop, sweets, and potato chips

*FV* fruit and vegetable, *WIC* Special Supplemental Nutrition Program for Women, Infants & Children, *SNAP-Ed* Supplemental Nutrition Assistance Program-Education, *EFNEP* Expanded Food and Nutrition Education Program

**Table 5:** Associations between discordance in dyad-reported food security status and household environmental factors compared to dyad-reported concordant food security status (88 Latino dyads)

	Odds ratio [95% confidence interval]
Acculturation score group (ref; concordant)	2.4 [0.8, 7.0]
Ever participated in nutrition education^a^ (ref; concordant)	3.4* [1.1, 10.0]
Home FV accessibility (ref; concordant)	3.1* [1.0, 9.4]
Cox-Snell R^2^	0.13

^a^ SNAP-Ed, EFNEP, WIC, and Cooking Matters; * *p* < 0.05

*FV* fruit and vegetable, *SNAP-Ed* Supplemental Nutrition Assistance Program-Education, *EFNEP* Expanded Food and Nutrition Education Program, *WIC* Special Supplemental Nutrition Program for Women, Infants & Children

## Discussion

The prevalence of household food insecurity was reported more often by Latina mothers compared with Latino fathers. Food security status was associated with neighborhood safety in both fathers and mothers, and with household income and home FV availability in fathers and mothers, respectively. Dyad discordance in reporting food security status occurred in 24% of the dyads and was related to dyad differences in reported frequencies of ever participated in nutrition education and perceived home FV accessibility.

The association between neighborhood safety and food insecurity was observed in mothers and fathers, providing evidence that the previous relationship observed between neighborhood safety and food insecurity among females [28] was also observed among males. Beliefs about neighborhood safety based on threats of crime or violence may increase perceptions of the difficulty of accessing resources [39], including those that influence food security such as community gardens, food pantries, or discount grocery stores. Therefore, in the current study, a perceived lack of neighborhood safety among both fathers and mothers may have been considered a barrier to accessing food resources, which contributed to assessment of food insecurity.

Household income is a well-known variable associated with food security status among both males and females [4, 12, 40]. The current study showed that low household income was related to reporting food insecurity only by fathers within dyads which may indicate that fathers have greater responsibility for household income as primary wage earners or that mothers may have less income information to consider [4, 41].

Low home FV availability was related to food insecurity only in mothers. This finding is consistent with two prior studies, one which observed a significant association between food insecurity and healthy food availability among a study population which was 86% female [26], and another which reported that food insecurity was associated with home FV availability among females [42]. Compared with fathers, mothers may be more aware of FV prices, which are generally more expensive than foods of lower nutritional value [43]. Mothers may also be more aware of the supply of FV at home based on meal planning and food preparation responsibilities, therefore more aware of how household food security status is related to home food availability than fathers. While fathers and mothers would share information about neighborhood safety, they might have different information related to foods, resulting in different factors associated with food security status.

Dyad discordance in reporting food security status was associated with discordance in reporting two variables, ever participated in nutrition education and home FV accessibility. Discordance in reporting participation in nutrition education was derived from the dyads where fathers had never participated in nutrition education while mothers had participated, rather than the dyads where mothers had never participated while fathers had participated. Participation in SNAP and SNAP-Ed were related to improvement in recipients’ food security status in other studies [21, 22]. Therefore, participation among fathers in such programs may provide fathers with food resource management skills, thereby improving home availability of healthy foods and stretching food dollars to address financial concerns, resulting in an increased number of dyads concordant in reported food security status as well as improvement in food security.

Concordance in reporting home FV accessibility was associated with concordance in food security status. Accessibility may provide visible reminders of FV availability to both fathers and mothers and support their common recognition of food security. Previous studies involving primarily White women [44] and Black, White and Hispanic women [27] reported an association between home food accessibility and food security, while the current study did not show an association between FV accessibility and food security among Latino fathers or mothers. The relationship between home food security and home food accessibility needs to be studied further by gender, healthfulness of foods being assessed for accessibility, and locations at home.

This study contained four main limitations. First, we used a 2-item food insecurity screening instrument to assess food insecurity, which is a limited measure of food insecurity. Hager et al. reported that the sensitivity and specificity of the 2-item food insecurity screen were 93 and 82%, respectively among caregivers of children in urban medical centers who were mainly Black (54%) and Hispanic (30%) [32]. Harrison et al. further reported that the sensitivity and specificity were 98 and 91%, respectively among adult general medicine outpatients who were mainly Black (51%) and White (42%) [45]. While the 2-item screening instrument was shown to be valid and acceptable in medical settings, the relationship between reports of food insecurity based on the 2-item food insecurity screen and the more comprehensive USDA 18-item Food Security module [1] needs to be evaluated among the Latino population. Second, the eligibility criteria for fathers included eating meals with the youth participants three times/week, whereas frequency of meals with the youth participant was not a criterion for eligibility for mothers. However, meal frequency with the youth participants at baseline was higher among mothers than among fathers. Third, low-income participants were recruited in a limited geographic area, which may not represent the Latino population in the U.S. Because the current participants were interested in a community-based intervention program to improve energy balance-related behaviors of their child, selection bias may have occurred. Finally, the question about participation in food assistance programs was based on current participation, while the food insecurity screen asked about conditions over the past year. Therefore, the perceptions of participants regarding food insecurity may not have matched the timeframe when fathers and mothers were participating in food assistance programs.

## Conclusions

Among Latino fathers and mothers, three factors were associated with food security including neighborhood safety in both fathers and mothers, household income only in fathers, and home FV availability only in mothers. Differences in associated factors may be dependent on fathers vs. mothers having access to information about different household conditions related to food insecurity. Dyad discordance in reporting food security status was related to dyad differences in frequencies of ever participated in nutrition education and perceptions of home FV accessibility. Nutrition education for fathers may improve dyad discordance in assessment of food security because nutrition education may improve awareness of home food supplies, which may influence perceptions of adequacy. Further research on relationships between food security and maternal perceptions of home food accessibility is needed.

## Data Availability

The data used in the current study are available from the corresponding author on reasonable request.

## Abbreviations

BMI: Body mass index
FV: Fruit and vegetable
SD: Standard deviations
*OR*: Odds ratio
CI: Confidence interval
NHANES: National Health and Nutrition Examination Survey
WIC: Special Supplemental Nutrition Program for Women, Infants & Children
SNAP-Ed: Supplemental Nutrition Assistance Program-Education
EFNEP: Expanded Food and Nutrition Education Program

## References

[1] Coleman-Jensen A, Gregory CA, Rabbitt MP. Food Security in the U.S. - Measurement. United States Department of Agriculture Economic Research Service. 2019. https://www.ers.usda.gov/topics/food-nutrition-assistance/food-security-in-the-us/measurement.aspx. Accessed 1 Sep 2020.

[2] Anderson SA (1990). Core indicators of nutritional state for difficult-to-sample populations. J Nutr.

[3] Foster JS, Adamsons K, Vollmer RL, Mobley AR (2019). A pilot study of low-income mothers and fathers of preschool age children to determine the relationship of food security and nutrition assistance on feeding style and child body weight. J Hunger Environ Nutr.

[4] Matheson J, McIntyre L (2014). Women respondents report higher household food insecurity than do men in similar Canadian households. Public Health Nutr.

[5] Wang K, Chu Y, Cuevas AG, Hasson RG, Tucker KL, Falcón LM (2018). Acculturation and food insecurity among Puerto Ricans living in Boston. J Nutr Educ Behav.

[6] Foster JS, Schwartz MB, Grenier RS, Burke MP, Taylor EA, Mobley AR (2019). A qualitative investigation into the U.S. Department of Agriculture 18-item household food security survey module: variations in interpretation, understanding and report by gender. J Public Aff.

[7] Coates JC, Webb P, Houser RF, Rogers BL, Wilde P (2010). “He said, she said”: who should speak for households about experiences of food insecurity in Bangladesh?. Food Secur.

[8] Amorim M, Silva S, Severo M, Kelly-Irving M, Samorinha C, Alves E (2019). Husbands’ and wives’ discordant self-reports on couple-level variables: implications for data analysis. Porto Biomed J.

[9] Coleman-Jensen A, Rabbitt MP, Gregory CA, Singh A. Household Food Security in the United States in 2019, ERR-275. United States Department of Agriculture Economic Research Service. 2020. https://www.ers.usda.gov/publications/pub-details/?pubid=99281. Accessed 24 Mar 2021.

[10] Myers CA, Mire EF, Katzmarzyk PT (2020). Trends in adiposity and food insecurity among US adults. JAMA Netw Open.

[11] Asfour L, Natale R, Uhlhorn S, Arheart KL, Haney K, Messiah SE (2015). Ethnicity, household food security, and nutrition and activity patterns in families with preschool children. J Nutr Educ Behav.

[12] Mazur RE, Marquis GS, Jensen HH (2003). Diet and food insufficiency among Hispanic youths: acculturation and socioeconomic factors in the third National Health and nutrition examination survey. Am J Clin Nutr.

[13] Hernandez DC, Reesor LM, Murillo R (2017). Food insecurity and adult overweight/obesity: gender and race/ethnic disparities. Appetite..

[14] Wright BN, Tooze JA, Bailey RL, Liu Y, Rivera RL, McCormack L, Stluka S, Franzen-Castle L, Henne B, Mehrle D, Remley D, Eicher-Miller HA (2020). Dietary quality and usual intake of underconsumed nutrients and related food groups differ by food security status for rural, Midwestern food pantry clients. J Acad Nutr Diet.

[15] Flórez KR, Katic BJ, López-Cevallos DF, Murillo R, Cancel-Tirado D, Aponte-Soto L, Echeverria SE (2019). The double burden of food insecurity and obesity among Latino youth: understanding the role of generational status. Pediatr Obes.

[16] Salinas JJ, Shropshire W, Nino A, Parra-Medina D (2016). Food insecurity, not stress is associated with three measures of obesity in low-income, Mexican-American women in South Texas. Food Public Heal.

[17] Pan L, Sherry B, Njai R, Blanck HM (2012). Food insecurity is associated with obesity among US adults in 12 states. J Acad Nutr Diet.

[18] TED: The Economics Daily. 26.8 million Hispanics or Latinos in the U.S. labor force in 2016. United States Department of Labor 2017. https://www.bls.gov/opub/ted/2017/26-point-8-million-hispanics-or-latinos-in-the-u-s-labor-force-in-2016.htm. Accessed 5 Sep 2020.

[19] Perreira KM, Pedroza JM (2019). Policies of exclusion: implications for the health of immigrants and their children. Annu Rev Public Health.

[20] Carney PA, Hamada JL, Rdesinski R, Sprager L, Nichols KR, Liu BY, Pelayo J, Sanchez MA, Shannon J (2012). Impact of a community gardening project on vegetable intake, food security and family relationships: a community-based participatory research study. J Community Health.

[21] Kaiser L, Chaidez V, Algert S, Horowitz M, Martin A, Mendoza C, et al. Food resource management education with SNAP participation improves food security. J Nutr Educ Behav. 2015; 47(4): 374–378. E1. doi: 10.1016/j.jneb.2015.01.012. 10.1016/j.jneb.2015.01.012 25843204

[22] Nord M (2012). How much does the supplemental nutrition assistance program alleviate food insecurity? Evidence from recent programme leavers. Public Health Nutr.

[23] Rabbit MP, Smith MD, Coleman-Jensen A, Powers D (2016). Food security among Hispanic adults in the United States, 2011-2014. Food insecurity.

[24] Carlos Chavez FL, Hernandez DC, Harris GJ, Grzywacz JG (2017). Household food security discordance among Latino adolescents and parents. Am J Health Behav.

[25] Sano Y, Garasky S, Greder KA, Cook CC, Browder DE (2011). Understanding food insecurity among Latino immigrant families in rural America. J Fam Econ Issues.

[26] Poulsen MN, Bailey-Davis L, Pollak J, Hirsch AG, Schwartz BS (2019). Household food insecurity and home food availability in relation to youth diet, body mass index, and adiposity. J Acad Nutr Diet.

[27] Nackers LM, Appelhans BM (2013). Food insecurity is linked to a food environment promoting obesity in households with children. J Nutr Educ Behav.

[28] Sanjeevi N, Freeland-Graves J, Hersh M (2018). Food insecurity, diet quality and body mass index of women participating in the supplemental nutrition assistance program: the role of intrapersonal, home environment, community and social factors. Appetite..

[29] Zhang Y, Reyes Peralta A, Arellano Roldan Brazys P, Hurtado GA, Larson N, Reicks M (2020). Development of a survey to assess Latino fathers’ parenting practices regarding energy balance–related behaviors of early adolescents. Health Educ Behav.

[30] National Center for Health Statistics. National Health and Nutrition Examination Survey (NHANES) 2015-2016 Anthropometry procedures manual. Centers for Disease Control and Prevention 2016. https://wwwn.cdc.gov/nchs/data/nhanes/2015-2016/manuals/2016_Anthropometry_Procedures_Manual.pdf. Accessed 1 Sep 2020.

[31] Kandula NR, Diez-Roux AV, Chan C, Daviglus ML, Jackson SA, Ni H, Schreiner PJ (2008). Association of acculturation levels and prevalence of diabetes in the multi-ethnic study of atherosclerosis (MESA). Diabetes Care.

[32] Hager ER, Quigg AM, Black MM, Coleman SM, Heeren T, Rose-Jacobs R, Cook JT, de Cuba SAE, Casey PH, Chilton M, Cutts DB, Meyers AF, Frank DA (2010). Development and validity of a 2-item screen to identify families at risk for food insecurity. Pediatrics..

[33] Neumark-Sztainer D, Wall M, Perry C, Story M (2003). Correlates of fruit and vegetable intake among adolescents: findings from project EAT. Prev Med (Baltim).

[34] Hanson NI, Neumark-Sztainer D, Eisenberg ME, Story M, Wall M (2005). Associations between parental report of the home food environment and adolescent intakes of fruits, vegetables and dairy foods. Public Health Nutr.

[35] Robinson-O’Brien R, Neumark-Sztainer D, Hannan PJ, Burgess-Champoux T, Haines J (2009). Fruits and vegetables at home: child and parent perceptions. J Nutr Educ Behav.

[36] Fulkerson JA, Neumark-Sztainer D, Hannan PJ, Story M (2008). Family meal frequency and weight status among adolescents: cross-sectional and 5-year longitudinal associations. Obesity..

[37] Saelens BE, Sallis JF, Black JB, Chen D (2003). Neighborhood-based differences in physical activity: an environment scale evaluation. Am J Public Health.

[38] Cervantes RC, Padilla AM, de Snyder NS (1991). The Hispanic stress inventory: a culturally relevant approach to psychosocial assessment. Psychol Assess.

[39] Matheson FI, Moineddin R, Dunn JR, Creatore MI, Gozdyra P, Glazier RH (2006). Urban neighborhoods, chronic stress, gender and depression. Soc Sci Med.

[40] Lorenzana PA, Sanjur D (1999). Abbreviated measures of food sufficiency validly estimate the food security level of poor households: measuring household food security. J Nutr.

[41] McIntosh WA, Zey M (1989). Women as gatekeepers of food consumption: a sociological critique. Food Foodways.

[42] Nunnery DL, Labban JD, Dharod JM (2018). Interrelationship between food security status, home availability of variety of fruits and vegetables and their dietary intake among low-income pregnant women. Public Health Nutr.

[43] Darmon N, Drewnowski A (2015). Contribution of food prices and diet cost to socioeconomic disparities in diet quality and health: a systematic review and analysis. Nutr Rev.

[44] Atoloye AT, Durward C (2019). Being motivated by nutrition is associated with healthy home food environment of supplemental nutrition assistance program (SNAP) recipients. J Nutr Educ Behav.

[45] Harrison C, Goldstein JN, Gbadebo A, Papas M. Validation of a 2-item food insecurity screen among adult general medicine outpatients. Popul Health Manag. 2020. 10.1089/pop.2020.0183. 10.1089/pop.2020.0183 33021444

